# What next for behaviour change professional development in general practice? Insights from an environmental scan and workshops

**DOI:** 10.3399/BJGPO.2023.0187

**Published:** 2024-06-12

**Authors:** Bryce Brickley, Jenny Advocat, Tze Lin Chai, Mitchell Bowden, Elizabeth Rieger, Lauren Ball, Raeann Ng, Nilakshi Gunatillaka, Elizabeth Ann Sturgiss

**Affiliations:** 1 College of Medicine and Public Health, Flinders Health and Medical Research Institute, Flinders University, Darwin, Northern Territory, Australia; 2 School of Primary and Allied Health Care, Monash University Faculty of Medicine Nursing and Health Sciences, Clayton, Australia; 3 Australian National University Research School of Psychology, Canberra, Australia; 4 The University of Queensland, Centre for Community Health & Wellbeing, Brisbane, Australia

**Keywords:** behavioural change, primary health care, general practitioners, nurses

## Abstract

**Background:**

A key role of general practice professionals (that is, GPs, and general practice nurses [GPNs]) is to support patients to change behaviours. Traditional approaches to assisting patients with, and learning about, behaviour change have modest outcomes.

**Aim:**

To explore behaviour change with GPs and GPNs and the availability of related professional development (PD) opportunities.

**Design & setting:**

Multi-methods study comprising an environmental scan survey of behaviour change tools and PD opportunities, and online workshops with Australian GPs and GPNs.

**Method:**

Survey data were analysed using qualitative content analysis, informing the design of the workshops. Workshop data included observation, note-taking, and collaborative reflection, which were analysed thematically and synthesised with survey data.

**Results:**

The survey had 18 complete responses. For the two virtual workshops, workshop 1 had 16 participants and workshop 2 had eight participants. There was diversity in awareness of existing behaviour change tools and resources. Preferences for future tools and PD opportunities related to specific aspects of its design, content, activities, and delivery. The following three themes developed from the workshop data: recognising the importance of relationships; recognising the importance of continuity; and keeping context in mind. In the absence of tools and resources, GPs and GPNs discussed behaviour change as something that occurs best through a patient-centred alliance that is continuing, respectful, grounded in trust and an understanding of their patient, and prioritises patient autonomy.

**Conclusion:**

Future general practice behaviour change PD should support clinicians to ‘assist’ patients and recognise the social and contextual influences on behaviour.

## How this fits in

Supporting GPs and general practice nurses (GPNs) to facilitate behaviour change with priority patient populations is critical globally. This study adds to the understanding about the availability of behaviour change resources for GP and GPNs, and their preferences for behaviour change professional development (PD). Most study participants were aware of behaviour change resources and behaviour change PD. However, they called for future PD opportunities to be underpinned by patient-centred care, with consideration into the design, content and activities, language, and mode of delivery.

## Introduction

Behaviour change is crucial for preventative health care worldwide.^1,2^ In Australia, approximately 38% of the total burden of disease is preventable.^3^ A nutritious diet, physical activity, and reduced use of alcohol, tobacco, and other drugs can improve mental and physical health.^4^ GPs and GPNs are at the front-line of Australia’s health system.^5^ Approximately nine in 10 people see a GP each year who are commonly the first point of contact for supporting health.^5^

The Royal Australian College of General Practitioners (RACGP) has published guidelines (the *Red Book*)^4^ outlining the roles and responsibilities of general practice teams, including GPs and GPNs, in facilitating behaviour change.^4^ GPs’ focus on behavioural counselling and risk assessment, whereas GPNs’ key roles include educating patients, and conducting follow-ups.^4^ Despite this delineation of tasks, there is a lack of clarity and confidence among some practitioners regarding their responsibility for delivering behaviour change interventions.^6^ Some GPs and GPNs have exhibited a lack of confidence in their ability to influence patient behaviours.^7,8^ General practice constraints, such as a lack of time and resources, and consultation dynamics make behaviour change interventions challenging for GPs and GPNs. Moreover, complex societal determinants of health, such as income, housing, employment, and public health policy, may require specialist skills for priority populations.^9,10^

Common behaviour change consultation strategies in general practice include the following: the 5As framework;^11^ person-centred care;^12^ a strengths-based approach;^13^ the stages of change (transtheoretical) model;^14^ and the behaviour change wheel.^15^ The 5As framework ('ask, assess, advise, assist, and arrange' in Australia) has been described as *'the universal approach to practising the art of enhancing behaviour change*'.^11^ A recent qualitative study reported that general practice clinicians have diverse understandings of the 5As framework and apply it variably in practice.^6^ It is a model that can assist with understanding the process that occurs between a clinician and patient for behaviour change. Some professionals in general practice believe that the available strategies are only helpful for those learning the basics of supporting patients in behavioural change (that is, university students).^6^

Little is known about what, if any, behaviour change resources are available to GPs and GPNs, and if these differ in application with priority patient populations. Additionally, the preferences of GPs and GPNs for behaviour change education and PD are unknown. This study aimed to address these knowledge gaps through an international environmental scan and virtual workshops.

## Method

### Research questions

The research questions for this study were as follows:

1. What support is available for GPs and GPNs working with priority populations to provide high quality, evidence-based behaviour change consultations to patients?
2. What are the perspectives and preferences of GPs and GPNs for behaviour change education and PD?

Priority patients include people from low-income, rural, or culturally and linguistically diverse communities, and First Nations people, where the social and cultural determinants may impact health behaviours and service utilisation.

### Research paradigm, methodological overview, and study design

This multi-methods study was situated within the pragmatic paradigm,^16,17^ with a relativist perspective, prioritising a qualitative approach.^18^ Each phase informed the next. An international environmental scan survey^19^ explored existing behaviour change learning platforms; preferences for format and content; and needs for future PD opportunities. Two online workshops followed the analysis of the scan.

### Formative environmental scan

#### Purpose

The environmental scan survey aimed to identify resources, organisations, and tools assisting primary care professionals in learning about behaviour change and/or delivering behaviour change interventions. Environmental scan surveys are commonly used to supplement systematic reviews of the literature or conducted as a formal part of programme planning.^19,20^ This method identifies what is relevant in practice through wide-scope screening.^19,20^

#### Recruitment and sample

Participants were identified through an iterative process involving research team members and established professional networks. The research team included practising GPs, dieticians, a psychologist, academics, a social worker, and medical students. These researchers collaborated to develop a list of clinicians, leaders, researchers, advocates, and policy influencers in general practice and primary care whose contact email was publicly available. Potential participants were emailed the survey link, and encouraged to share it with their colleagues, allowing recruitment to snowball.

#### Survey design and data collection

A brief (10-minute) online survey (Supplementary Box 1) was collaboratively developed and hosted on LimeSurvey. All researchers participated in drafting the survey. Before delivery, the survey was pilot tested through group cognitive interviewing.^21^ The survey started with questions about the availability of behaviour change tools and PD resources. Then, participants responded to demographic questions such as sex, primary care role, and experience. The survey remained active for 7 weeks.

#### Data analysis

Open-text responses were extracted to an Excel spreadsheet and inductive qualitative content analysis was conducted to organise data into concepts reflecting a condensed, broad description of the results.^22^ Surveys without any responses to questions about behaviour change tools and PD opportunities were classified as incomplete and discarded. BB read and reflected on the survey data in the context of the research questions to prepare for organisation. The key concepts in each question informed the unit of analysis (that is, descriptions of PD organisations and behaviour change tools). After data familiarisation, BB organised the data into groups and developed codes that distilled the text groups into broad concepts. We employed qualitative content analysis specifically to develop broad descriptive concepts. All researchers debriefed and reflected on the evolved concepts and the data associated with each concept. This informed the design and development of the workshops.

### Virtual workshops

#### Design

Workshop 1 focused on behaviour change understandings and workshop 2 centred on reflections of a case study and preferences for the format of behaviour change PD opportunities. Both workshops used breakout rooms for smaller group discussions.

#### Recruitment and sample

Eligible participants were practising GPs and GPNs in Australia. Recruitment was conducted through advertisements on social media, researcher professional networks, and professional newsletters. Recruitment snowballed through professional networks. An AUD$150 e-gift card honorarium was provided.

#### Data collection

Data collection and analysis included observation, reflection, and discussion by the researchers. Both workshops (lasting around 90 minutes) had a maximum of 20 total participants, and at a minimum more GP and GPN participants than investigators. The workshops were recorded via a videoconferencing platform and saved to a password protected shared drive. We documented de-identified reflections and observations in a shared Google document.

Workshop 1 included a short reflection on behaviour change theories. This was followed by a facilitated breakout session to discuss behaviour change, including the implementation challenges, preference for learning about skills, key behaviour change topics for learning, and ideas for supporting resources. Participants rejoined the larger group to share stand-out ideas from their discussion.

Workshop 2 began with a recap along with a case study. This was followed by a facilitated discussion in breakout rooms where participants discussed their reflections. Participants rejoined the larger group and shared key reflections before the facilitator concluded the session.

#### Data analysis

Data analysis was iterative and started simultaneously with data collection. Team meetings were held to discuss, debrief, and reflect on the workshops. The researchers reflected on audio-recordings of the workshops and the audio-recordings were transcribed using Otter and reviewed by ES, JA, and TLC. Following an inductive approach, workshop data were coded, and codes were organised to develop initial themes (ES, JA, TLC). This process was supported by field notes from workshop facilitators. ES presented the themes to the broader investigator team at a team meeting in which the themes were discussed and finalised.

## Results

### Participants

#### Environmental scan

The environmental scan survey was sent to 345 participants and associations (Society for Academic Primary Care, World Organization of National Colleges, Academies and Academic Associations of General Practitioners/Family Physicians [WONCA], RACGP, and Australian Association for Academic Primary Care [AAAPC]) across five countries (Australia, New Zealand, UK, US, and Canada) between March 2022 and April 2022 (*n* = 18 complete responses; *n* = 28 incomplete responses that were discarded). All responders were from Australia (13 females, eight males), including practising GPs (*n* = 7), GP academics (*n* = 6), clinical governance manager (*n* = 1), healthcare manager (n=1), medical education content developer (*n* = 1), nurse educator (*n* = 1) and telephone counsellor (*n* = 1). All responders had tertiary education qualifications: post-graduate degree (*n* = 17) and undergraduate degree (*n* = 1).

#### Virtual workshops

Two 90-minute virtual workshops occurred on 27 April 2022 and 8 June 2022. A total of 30 participants registered to attend Workshops 1 and 2 combined. Workshop 1 had 16 participants (13 GPs, 3 GPNs), while workshop 2 had eight participants (two GPNs and six GPs), with seven participants having attended the previous workshop (workshop 1), and one new attendee. Illustrative quotations are provided below by GPs or GPNs.

### Availability of behaviour change tools and professional development opportunities

Environmental scan findings in Table 1 illustrated that healthcare professional organisations were frequently identified by participants to provide behaviour change PD opportunities (*n* = 11). Participants commonly identified behaviour change tools as programmes (*n* = 4) and frameworks (*n* = 11). Some participants were unaware of organisations providing PD opportunities (*n* = 4) or tools for behaviour change (*n* = 4).

**Table 1.** Environmental scan results: awareness of existing behaviour change tools and organisations that provide behaviour change professional development opportunities

Question	Category	Response, *n*
Are you aware of any organisations that provide professional development for workers wanting to change behaviour in others (this could be in industry such as health care, education, law)? If so, please list (**viable responses *n* = 14**)	**Unaware of organisations that provide professional development opportunities**	4
	**Professional organisations in health** Royal Australian College of General Practitioners Other professional or member-based organisations	11
	**Academia** Australian University	1
	**Independent, not-for-profit, or volunteer organisations in health** Volunteer organisations in health care Independent, not-for-profit organisations in health	5
Are you aware of any tools that exist for workers who strive to facilitate behaviour change in others? (**viable responses *n* = 33**)	**Unaware of tools that exist for workers**	4
	**Aware of tools that exist for workers, however, unable to describe**	2
	**Clinical guidelines** Royal Australian College of General Practitioners	3
	**Behaviour change programmes** Online Unspecified if online or face-to-face	4
	**Behaviour change frameworks** Consultation frameworks Change management frameworks	11
	**Learnt interventions** Psychological therapy	1
	**Medicare funded** Physician-led Nurse-led	3
	**Decision support tools** Patient decision aids Risk-assessment tools	2
	**Formal tertiary education** Bachelor level	1

Workshop participants noted a lack of availability of PD opportunities about behaviour change. One participant said:

*'There’s not a flood of offers for that sort of training, especially now. We used to have a better set up with divisions of general practice … to support more education, specifically, and supporting practice nurses.'* (GPN1)

### Preferences for behaviour change tools and professional development

Environmental scan findings in Table 2 showed participant preferences for PD opportunities in design (*n* = 13) (for example, time, remuneration, access), content and activities (*n* = 12) (for example, resources, language, small group learning), and delivery (*n* = 4) (for example, professionalised, patient-led). Similar aspects were identified as important to make behaviour change tools most useful: design (*n* = 21), content (*n* = 6), and delivery (*n* = 5).

**Table 2.** Environmental scan results: preferences for new behaviour change tools and behaviour change professional development opportunities

Question	Category	Responses, *n*	
If we were to create a behaviour change professional development opportunity, what should we consider to make it most useful to you? (**viable responses *n* = 34**)	**Modality** Online Online and face-to-face	5	
	**Design** Asynchronous Free or easy to access Easy to use Brief Logical to work through Evidence-based Applicable to broad range of behaviours or conditions Advanced training options Appropriately remunerated for the professional PD opportunities for GPs should be tailored towards the individual doctor and their patient profile	13	
	**Content and activities** Effective audio-visual resources Concise, unambiguous language Small group reflection Opportunity for reflective writing Helping the professional measure success PDF summary Webinar Modelling language Understanding deeper motivations Trust and therapeutic relationship A range of behaviour change examples	12	
	**Delivery** Professionalised Incorporated into routine practice or existing systems Can be patient-led and started outside consultation hours	4	

If we were to create a behaviour change tool, what should we consider to make it most helpful to you? (**viable responses *n* = 32**)	**Design** Free or easy to access Easy to use Versatile Brief Evidence-based Practical Patient acceptability	21	
	**Content** Culturally sensitive Gender-sensitive Easily understood Complementary patient and provider material Patient-centric Diversity	6	
	**Delivery** Incorporated into routine practice or existing systems Ongoing evaluation	5	

PD = professional development

Workshop participants noted their interest in PD linked to specific topics, so that clinicians who had a specific interest (for example, obesity) could be exposed to relevant learnings. One participant noted that behaviour change is *'so deeply embedded in everything that we need to do as GPs'* (GP3).

Barriers to attending PD included lack of paid time for nurses, which was seen as costly, and the need for their role to be covered in their absence. One GPN talked about using YouTube:

*'I'll just kind of self-research that stuff … but it’s not formal.*' (GPN1)

GPs echoed this approach. Participants spoke about relying on skills they picked up informally.

*'I think with the behaviour change aspect, I'm using the skills that I've learnt elsewhere, that actually, I don't really look for resources …'* (GP2)

Others had a preference to learn in an interactive format or from a mentor, in person, rather than through reading. One participant said:

*'... it’s quite useful to see someone do it well, then, obviously, you do your best to emulate it.*' (GP3)

### Themes from the virtual workshops

The following three main themes, which relate to successful behaviour change consultations, were developed from the analysis of the workshop data: (1) recognising the importance of relationships; (2) recognising the importance of continuity; and (3) keeping context in mind. These themes reflected participants views on core topics for future behaviour change education and PD to support GPs and GPNs working with priority patient populations.

#### Theme 1: Recognising the importance of relationships

##### Building trust and focusing on patient goals

Effective clinician–patient relationships were felt to contribute to successful behaviour change consultations. Participants viewed a trusting clinician–patient relationship as important to facilitate behaviour change. It was thought that by building trust and rapport with patients, clinicians would be better placed to understand patient needs and influence their readiness to change behaviours. Participants reported that once trust is established, then clinicians can tailor behaviour change communication based on patients’ priorities, values, and motivations:

*'… That’s the key, developing that relationship and building that trust to then explore with them what are they wanting to do.'* (GP4)

General practice clinicians spoke of drawing on their relationship with patients, and approaching behaviour change through a strengths-based framework that is responsive to patient circumstances, values, and goals:

*'…* [it] *is about relationship, which is about curiosity, respect, being with the person where they’re at. And through that you can sometimes start to understand what their values are … what’s important to them, what they have the confidence to do, and what they want to change when they are ready to do that.'* (GP5)

One GP participant expressed the importance of patient autonomy and patients leading goal setting:

*'It’s about the person. It’s about the patient. It’s their life. They have to buy-in and they have to drive it … You can't achieve anything with patients unless they're actually willing to participate.*' (GP4)

Participants acknowledged that providing behaviour change support may require a sensitive approach. Raising the topic of behaviour change at an inappropriate time runs the risk of negatively impacting that relationship, making it impossible to address behaviour change needs:

*'You’ve got someone, they know* [smoking is] *bad for them, and that you think that they should quit. But … if they said they’re not even interested or open to that, you’re actually just going to alienate them more, or get them offside or disrupt the therapeutic alliance by talking to them about it. And it will come across as more criticism or judgement … You’ve got to build that space where they are receptive first.'* (GP4)

##### Leading team-based care

In addition to the clinician–patient relationship, team-based care was thought to contribute to successful behaviour change consultations. Participants highlighted the importance of patients building connections with other health professionals. As patients often see a variety of healthcare professionals, incorporating behaviour change as part of GP-led, team-based care can be useful to assist patients with behaviour change:

*'It’s acknowledging the practice team and the skills to support someone. It was acknowledging that we have that capacity within that practice and let’s actually utilise those skills and feedback — good feedback mechanisms with the patient and the GP who is managing the care of that patient.'* (GPN1)

*'Recognising that it is never a one-off conversation, it’s often not just one practitioner that will make the change. That … the broader discussion and having support from multiple people can make a big difference.'* (GP4)

### Theme 2: Recognising the importance of continuity

Continuity was viewed as enhancing understanding of patient perspectives, improving capability to deliver long-term behavioural support, and in some cases enabling clinicians to incorporate the creative use of failure into the process of behaviour change. These relationships were thought to facilitate a better understanding of factors in a patient’s social environment and history:

*'It’s a longitudinal relationship. If we’re lucky, we know them, their families, their communities.'* (GP5)

Through the provision of longitudinal care, clinicians can consider longer-term outcomes. Thus, participants reported that behaviour change should include long-term support:

*'I want to play a long game with this. I’m wary of things that set them up for short-term solutions that don’t last …'* (GP6)

Continuity was discussed as having an impact on how behaviour change consultations unfold and what they can achieve. Participants expressed that any activities to encourage behaviour change should occur at multiple points throughout the care continuum:

*'We can have these* [behaviour change] *conversations over time. We have these longitudinal relationships …*[so] *that we don’t have to get it all done in one go.'* (GP5)

*'And then each session, it might be weekly, you get him to feel safe and trusted. And you take it really slowly.'* (GP7)

Clinicians said timing mattered when bringing up the topic of behaviour change. Assessing the patient’s willingness and readiness for behaviour change at each consultation can improve the clinician’s ability to support the behaviour change process:

*'Being there for the time when change is more likely to be something that the patient is planning is helpful.'* (GP8)

#### Behaviour change and ‘failure’

Some participants highlighted the fact that behaviour change is a process and recognised that it is the role of the clinician to support patients to try again when they fail:

*'If you look at change as a singular event as opposed to a process, obviously* [for] *any single attempt the most likely outcome is failure … fundamentally it’s a process.'* (GP9)

One participant described using ‘failure’ as a way to better understand the patient’s needs:

*'If they come back and they say, "I couldn’t do it, Doc." I’d go, "Okay, I’m curious what happened. What got in the way?" It’s that growth mindset, so if it doesn’t work; let’s revisit it, let’s do it again …'*

### Theme 3: Keeping context in mind

Participants discussed context in two ways: the care model and patients’ lives. Primary care models, such as private practices or community health centres, and funding mechanisms were thought to influence clinical capacity for behaviour change. Specifically, private general practice business models, in which patients pay fees for services, may influence whether behaviour change is raised:

*'The same sort of dialogue would have been much less acceptable when I was working in private general practice. It is important that we recognise there is a unique element to the context of … general practice, where it really is for the long term.'* (GP9)

One participant working in community health articulated one of the ways that the model of care can influence a behaviour change consultation:

*'It’s a community service and fee-free for the clients, it’s about the power dynamic. For a lot of the people that are seeing me, they can’t go somewhere else, or they have before, and it didn’t work. And you can almost be a bit firmer.'* (GP4)

Participants discussed context-based challenges for facilitating behaviour change in primary care, such as the impact of poor remuneration on time, high patient demand or caseloads, and competing pressures. Limited time in consultations was thought to result in behaviour change being de-prioritised below acute needs:

*'Part of the problem is the short consults in general practice … We don’t get remunerated for doing good medicine. There’s a big systemic issue. They need to get rid of all the paperwork and the documents. I’m worried about being audited that we’re not doing it properly and just let us do good medicine.'* (GP7)

Participants discussed how the context of patients’ lives may impact behaviour change consultations and how the focus on the individual patient obscures social factors that may have a much larger impact on a patient’s capacity to change:

*'The individualisation of a problem doesn’t help — we have an individual solution as though it’s an individual problem, when it’s actually a social problem. If we can get rid of half the advertising for sugary things that might make more difference for our communities than teaching GPs these processes. It’s almost individualising the health response.'* (GP6)

## Discussion

### Summary

This study contributes new evidence around behaviour change in general practice. Overall, most, but not all participants were aware of (i) behaviour change tools for use among priority patient populations, and (ii) organisations who provide behaviour change PD opportunities. In the absence of tools and resources, GPs and GPNs in our study reported that behaviour change is best achieved through a patient-centred alliance that is continuing, respectful, grounded in trust and an understanding of their patient, and prioritises patient autonomy. These components are inherent to the philosophies of best practice for GPs and GPNs and are thought to drive behaviour change efforts in general practice. Priorities for future training and research include the following: (i) increasing the availability of evidence-based tools and PD opportunities; (ii) increasing support for general practice clinicians to achieve the 5As,^11^ especially 'assist'; and (iii) developing new approaches to navigate the powerful social influences of behaviour and facilitate behaviour change within the constraints of general practice.

### Strengths and limitations

The patient perspective was out of scope of the present study, emphasising the need for future research to include patient perspectives informing GPs and GPNs behaviour change PD. There were few viable responses to the environmental scan (*n* = 18); less than one-third of the sample were practising GPs and GPNs; and there was no engagement by participants outside of Australia. Participants were asked to forward the survey link through their networks, although the extent to which this occurred is unknown owing to the nature of online surveys.^23^ Despite the survey being pilot tested before use with general practice clinicians, 60% of accesses to the survey resulted in a complete lack of response to the questions about behaviour change tools and PD opportunities, and were therefore discarded. Inability to capture the motives of those who did or did not complete the survey is a known limitation of the online survey method.^23^ The workshop participants were weighted towards GPs, however, workshop facilitation and the use of breakout rooms was a strength of the work and improved equity of engagement.

While a sample of general practice clinicians who are interested in behaviour change introduces bias, it led to higher levels of engagement during the case-based workshops. This is a strength of the workshops because it resulted in creating '*shared meaning and joint action*' (a core focus of pragmatism),^17^ and this was particularly evident when sharing breakout-room ideas with the larger group.

The participatory approach minimised the distance and separateness between researchers and participants. This closeness is valuable for fostering networks among general practice stakeholders involved in research, a crucial factor in developing leaders in general practice research.^24^ However, the close proximity and relationships between researchers and participants posed a risk of bias in the interpretive findings by introducing the views and experiences of the researchers in the workshops. Researchers were aware of this position and implemented mitigation strategies. Researchers strived to be open facilitators, encouraging in-depth discussions among participants while maintaining a degree of separateness from the data.

### Comparison with existing literature

This study, aligning with a recent global literature review, identified a lack of behaviour change tools for use with priority patient populations.^25^ Participants tended to recognise health professional organisations (for example, RACGP) as providers of behaviour change PD, although some participants were unaware of any existing tools and resources, with an explicit lack of tailored support for GPNs. These findings suggest that evidence-based tools and behaviour change education opportunities are currently underutilised in general practice, and this is a persistent theme across medical education.^26^ Increasing the availability of evidence-based tools and education opportunities, and increasing their accessibility by consolidating them, will help to better support general practice clinicians and improve patient outcomes.

In Australia, general practice features a diverse workforce serving varied populations across a range of contexts.^5^ Study participants acknowledged that they work with individuals and families whose behaviours are influenced by complex societal factors, such as advertising, and social and cultural determinants. Participants warned against the individualisation of behaviour in general practice PD and clinical practice, and called for a greater emphasis on recognising context. These powerful contextual factors that were outside of the GP's control were perceived to make the use of behaviour change tools and PD opportunities less relevant. More research is needed to understand and respond to these powerful contextual factors in general practice. General practice research is currently underfunded, particularly in rural and remote areas where health outcomes are notably poorer than urban areas.^27^ Equitable funding is needed to enable research and evaluation in areas where ill-health behaviours are prevalent and care services are limited, such as rural and remote health contexts.

### Implications for research and practice

Participants relied on their individual strengths to inform behaviour change efforts, rather than searching for tools and additional PD opportunities. They initiated behaviour change efforts based on strong relationships (with patients and the clinical team), and self-confidence in clinical skills to use the 5As,^11^ especially ‘ask’ and ‘assess’. However, we observed a gap in participants’ confidence to build on their strengths and actively support patients towards change (that is, 'assist and arrange’). A recent content analysis of the *Red Book*, using the 5As, found that mentions of ‘ask and assess’ (*n* = 102) were disproportionately higher than ‘assist and arrange’ (*n* = 34).^28^ This gap was more pronounced in the preventative care guidelines for Aboriginal and Torres Strait Islander people (‘ask and assess’ *n* = 259 versus ‘assist and arrange’ *n* = 22).^28^ Survey responses identified clinical guidelines and tools as available behaviour change resources, stressing the importance of language in resources. To enhance general practice clinicians’ strengths and support behaviour change efforts, future resources should encompass all 5As, including the ‘assist’ and ‘arrange’ components.

Participants infrequently described strategies to ‘assist’ patients, focusing on delivering behaviour change efforts at various stages across the care continuum to nurture clinician–patient relationships. A sample of 2010 GPs who entered in the Australia-wide RACGP Alcohol and Other Drugs GP Education Programme were least confident to ‘assist’ patients compared with other 5As components.^29^ In practice, general practice clinicians have been less successful in ‘assisting’ patients and ‘arranging’ care.^30^ This is critically important as care that includes the ‘assist’ component is more likely to lead to changes in patient behaviour.^31^ Future education opportunities should build on general practice clinicians’ strengths by emphasising skills, confidence, and knowledge around the ‘assist’ aspect of facilitating behaviour change.

In conclusion, this study has provided new insights into understanding behaviour change in general practice. Increasing the availability and accessibility of evidence-based tools and behaviour change education opportunities in general practice is needed. When developing new behaviour change resources, consideration should be given to the design, content and activities, and mode of delivery. Future general practice behaviour change PD should build on GP clinicians’ strengths by supporting them to ‘assist’ patients, avoiding the individualisation of behaviours and consider the contextual influences on behaviour.

## Data

The authors confirm that the data supporting the findings of this study are available within the article.
